# Dynamic Balance Measurement and Quantitative Assessment Using Wearable Plantar-Pressure Insoles in a Pose-Sensed Virtual Environment

**DOI:** 10.3390/s18124193

**Published:** 2018-11-30

**Authors:** Cunguang Lou, Chenyao Pang, Congrui Jing, Shuo Wang, Xufeng He, Xiaoguang Liu, Lei Huang, Feng Lin, Xiuling Liu, Hongrui Wang

**Affiliations:** 1 College of Electronic Information Engineering & Hebei Key Laboratory of Digital Medical Engineering, Hebei University, Baoding 071002, China; loucunguang@163.com (C.L.); pancheyapcy@sina.com (C.P.) congrui_jing@163.com (C.J.); wangshuo5970@sina.com (S.W.); xufeng_he@163.com (X.H.); xiaoguang8310253@163.com (X.L.); asflin@ntu.edu.sg (F.L.); 2 Department of Molecular, Cell and Cancer Biology, University of Massachusetts Medical School, Plantation Street, Worcester, MA 01605, USA; lionishystone@gmail.com; 3 School of Computer Science and Engineering, Nanyang Technological University, Singapore 639798, Singapore

**Keywords:** plantar pressure, pressure insole, Kinect, balance estimation, virtual reality

## Abstract

The center of plantar pressure (COP) reflects the dynamic balance of subjects to a certain extent. In this study, wearable pressure insoles are designed, body pose measure is detected by the Kinect sensor, and a balance evaluation system is formulated. With the designed games for the interactive actions, the Kinect sensor reads the skeletal poses to judge whether the desired action is performed, and the pressure insoles simultaneously collect the plantar pressure data. The COP displacement and its speed are calculated to determine the body sway and the ability of balance control. Significant differences in the dispersion of the COP distribution of the 12 subjects have been obtained, indicating different balancing abilities of the examined subjects. A novel assessment process is also proposed in the paper, in which a correlation analysis is made between the de facto sit-to-stand (STS) test and the proposed method; the Pearson and Spearman correlations are also conducted, which reveal a significant positive correlation. Finally, four undergraduate volunteers with a right leg sports injury participate in the experiments. The experimental results show that the normal side and abnormal side have significantly different characters, suggesting that our method is effective and robust for balance measurements.

## 1. Introduction

Balance ability is an important physiological function of human body, and it plays an irreplaceable role in maintaining the normal posture and activity of human body [1,2,3]. Balance also represents coordinating function for the trunk muscles and joints, especially hips, knees, and ankles [4]. Muscle dysfunction and weak core strength can lead to poor balance performance, which can result in incorrect walking posture and even sports injuries. Aging, stroke, physical disability, and other factors will cause muscle dysfunction and thus lead to decline of human body balance ability, which is commonly considered as a risk factor for falls and fall-related injuries in older adults [5]. Therefore, the assessment of balance ability plays a pivotal role in the diagnosis of physical diseases.

In literature, the balance scale has been served as the assessment method in clinics and research. The balance performance of subjects can be obtained using the scores when they perform the corresponding actions. The sit-to-stand (STS) test has been used as a measure of lower-limb strength in older people and those with significant muscular weakness [6]. Recent studies suggest that performance in the STS test is influenced by factors that are associated with balance and mobility [6]. Whitney S.L., et al. has proved that the data from the sitting posture test can reflect the balance of patients with balance disorders [7].

The force measuring platform and 3-D motion capture system have been employed in laboratory studies of postural stability [8,9,10]. To maintain an upright stance, the center of mass (COM) should be stable and controllable [11,12,13]. Cherng R.J., et al. have found that the COM could explain the degree of body shaking, which is related to the balance ability and risk of falls [13]. However, due to the inhomogeneous density distribution of human body, it is difficult to accurately measure the COM [14]. The ability to maintain the center of pressure (COP) within the base of support can also estimate the balance ability well [11,15,16,17]. Tomoya T., Abby M., et al. have used COP for balance assessing and rehabilitation training [18,19]. Kawano T., et al. have used the COP and surface electromyography to formulate the degree of postural instability and concluded that postural instability increased exponentially with the COP displacement [20]. In addition, there is a significant effect of sway amplitude and direction on the measurement error of COP, with an increase in error as sway amplitude increases and a significantly accumulated error in the sway direction [21]. These results show that the COP can reflect the state of motion, and the shift speed of COP provides valuable information for the assessment of human balance ability.

Compared to other pressure measurement systems, pressure insoles can effectively collect data during a variety of activities and the wireless communication technology provides much more flexible data acquisition [22,23,24,25,26]. Moreover, the wearer is under the natural state without inconvenience and discomfort [22]. For an integrated solution, the Kinect sensor enables effective human-computer interaction utilizing its motion capture technology. However, it is still difficult to estimate the balance activity accurately if the Kinect sensor is not used in connection with the other COP and COM devices [4,27,28,29].

In view of the pros and cons of the existing technologies, we propose a novel dynamic balance evaluation system, combining the functions of the Kinect sensor and wearable pressure insoles. The following major contributions are made: (i) The subjects can undergo the Kinect interaction and plantar pressure data acquisition at the same time. (ii) When the subject’s body leans forward to the designed degree, the Kinect interaction can be judged as standard and then the subject can pick the apples with upper limb movement while collecting the plantar pressure. (iii) The COP can be calculated from the plantar pressure data, and the COP displacement is employed to assess the balance ability of the subjects. (iv) In order to verify the availability of the balance estimation method, the STS test is conducted for 12 subjects and the COP displacement is extracted for analysis. The results of the two experiments have been analyzed using the Pearson and Spearman correlation analyses. (v) Finally, the balance of four abnormal subjects are assessed by the proposed balance assessment system to further verify its effectiveness.

## 2. Methods

### 2.1. Experiment System

**Figure 1:**
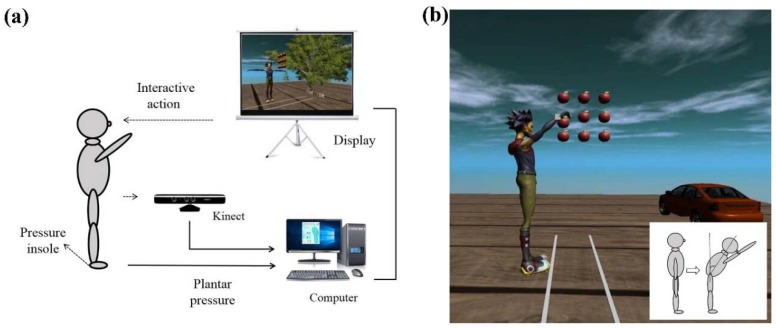
(**a**) Schematic drawing of the designed balance assessment system; (**b**) Kinect interaction interface. The inserted figure shows the schematic of the forward leaning of the body.

The designed system consisted of a Microsoft Kinect sensor, pressure insoles, a personal computer (PC) and a display screen, as shown in Figure 1a. The Kinect sensor was preconditioned and calibrated before use. The PC received, archived, and processed the data. The interactive virtual scene was visualized instantly with the help of the Kinect sensor and its toolkits. In this study, an apple picking game was designed as shown in Figure 1b, and the inserted figure shows the schematic of the forward leaning of the body.

The Kinect sensor is a widely used color-depth (RGB-D) camera that can capture human motion in real-time via Kinect, and it can give a pose action interactive screen to guide the subject to follow. In combination, the developed pressure insole is a pair of equipment that measures and acquires the foot plantar pressure and transmits the data via WiFi. The insoles have 28 sensing elements with a sampling rate of 50 Hz for each element, hence 1400 foot pressure data points can be collected per second. The Tekscan pressure pad (Tekscan BPMSTM, Inc., South Boston, MA, USA) was employed in the measurements for verifying the plantar insole’s effectiveness. The Tekscan sensors are 0.1 mm thin and contain 572 individual sensor-elements (62 per cm^2^), which are oriented along 26 rows and 22 columns. Each sensor covers a measurement area of 22 mm × 33 mm, with a resulting spatial resolution per sensel of 1.02 mm × 1.27 mm.

### 2.2. Experiment Procedure

Twelve healthy university student volunteers (ages 24.0 ± 1.0, height 178.0 ± 5.3 cm) and four volunteers with right leg sports injury (2 males with ages 21, height 176.5 ± 1.5 cm and 2 females with age 20.0 ± 0.5, height 157.0 ± 1.0 cm) were recruited to participate in the experiment. The twelve healthy university student volunteers had no muscle or nerve diseases related to balance disorders, and were requested to wear loose clothes. The four abnormal volunteers had different degrees of right leg injuries, but they can be stressed at full foot. All participants were informed about the contents of the experiment and signed an informed consent form.

**Figure 2:**
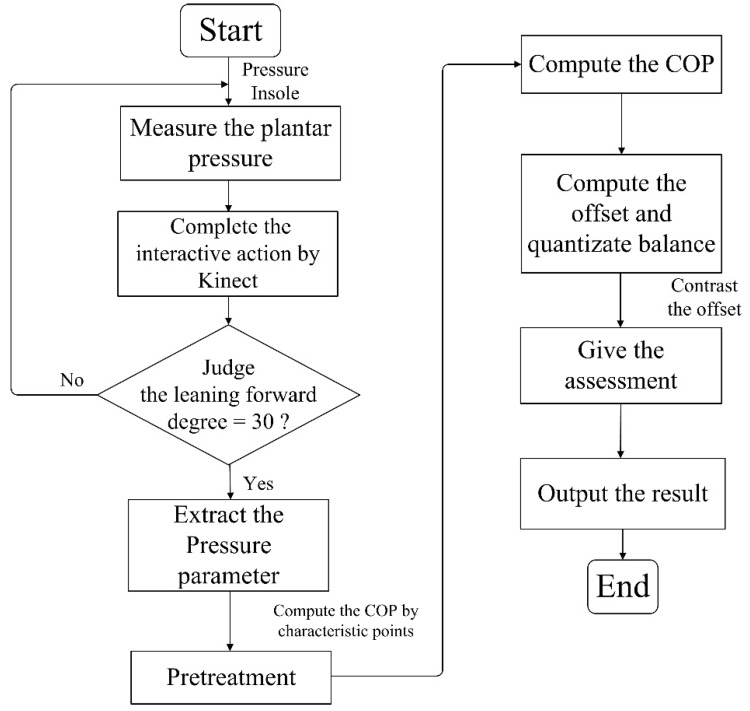
The flowchart of Kinect guided balance measurement.

To validate the effectiveness of the insole pressure system, the gait data were acquired and compared with the Tekscan system. One subject stood naturally upright on the pressure pad, wearing the pressure insoles and hanging his hands on both sides of his body. After that, they were to adjust their feet to the same width as their shoulders, without moving or turning their head, and to keep their eyes looking forward. The flowchart of the experiment procedure is shown in Figure 2. A time cost of 30 s was required per experiment and each experiment was repeated at least three times. Before performing the experiment, the subjects wore the pressure insoles and stood within the identifiable range of the Kinect sensor. In the process of human interaction, the feet were not moved. After the volunteers got familiar with the process of the Kinect interaction, each subject was requested to perform the experiment until 1500 frames of valid data were obtained. Valid data were the plantar pressure data that were collected when the body leant forward to the designed angle (30 degree in the experiment). The pressure data were preprocessed on PC with de-noising, then the COP was calculated and the maximum COP displacement within 30 s was taken for analysis.

**Figure 3:**
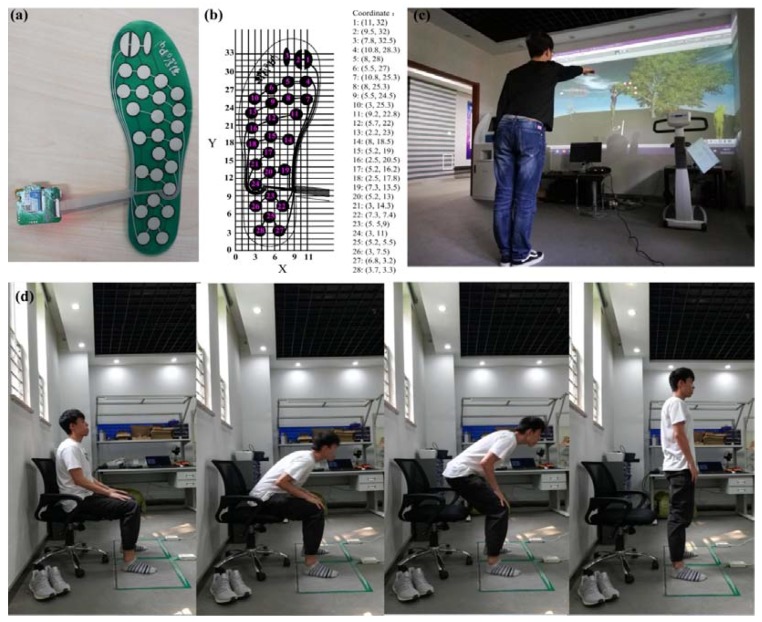
(**a**) Plantar pressure insoles; (**b**) pressure insoles coordinate system; (**c**) Kinect interactive scene; and (**d**) the sit-to-stand (from left to right) process.

The developed pressure insoles are shown in Figure 3a. The coordinate system of the insoles was set up and used to calculate the COP, as shown in Figure 3b. The Kinect sensor guided the subject to perform an action of picking apples displayed on screen while keeping the body leaning forward, as shown in Figure 3c. The COP displacement and the shift speed of COP were calculated for analysis. Subsequently, the sit-to-stand (STS) test was performed as shown in Figure 3d. Each subject sat on a chair with the same height as their knee, the feet were apart parallel to one another with the same as the humeral ministry wide. When the start command was issued, the subject stood up from the chair and then sat down, repeating the action continuously for 30 s. The experiment should be repeated at least three times, the plantar pressure data of each experimental subject was recorded and the maximum COP displacement was taken as the analysis data. Finally, the feasibility and effectiveness of the proposed method was verified by comparing with the results obtained using the STS tests.

### 2.3. Data Processing

The pressure insoles were connected to the PC through Wi-Fi. The pressure insole transmits 28 raw data points per frame to the PC in real time. According to the design principle of the circuit schematic, the pressure value of each sensor element $F_{i}$ can be calculated using the formula:

(1) $$F_{i}=\frac{{4096-x}_{i}}{x_{i}}\times m\;(i=1,2,3,\ldots ,28)$$

where $x_{i}$ is the captured raw data from each sensor element, and m is an adjustment parameter, which is determined to be 2000 according to the calibration of actual force value.

We set up a coordinate system for the insoles and the coordinates of each sensor element were obtained. Then, the COP coordinate, COP lateral displacement, COP longitudinal displacement, COP displacement, and the shift speed of COP can be calculated through the following equations [22]:

(2) $$X_{\mathrm{COP}}=\frac{\sum_{i=1}^{28}X_{i}F_{i}}{\sum_{i=1}^{n}F_{i}}$$

(3) $$Y_{\mathrm{COP}}=\frac{\sum_{i=1}^{n28}Y_{i}F_{i}}{\sum_{i=1}^{n}F_{i}}$$

(4) $${\mathrm{LateralD}}_{\mathrm{COP}}{=X}_{\mathrm{COP}}\left(t\right)-X_{\mathrm{COP}}(t-1)$$

(5) $${\mathrm{LongitudinalD}}_{\mathrm{COP}}{=Y}_{\mathrm{COP}}\left(t\right)-Y_{\mathrm{COP}}\left(t-1\right)$$

(6) DisCOP=(LateralDCOP2+LongitudinalDCOP2)12

(7) $${\mathrm{Speed}}_{\mathrm{COP}}=\frac{1}{\Delta t}\overset{t+\Delta t}{\underset{t}{\sum }}{\mathrm{Dis}}_{\mathrm{COP}}$$

where the ith sensor element is defined by the $X_{i}$ and $Y_{i}$ coordinates, $X_{\mathrm{COP}}$ and $Y_{\mathrm{COP}}$ are the COP coordinates, t(s) is the current time, ${\mathrm{LateralD}}_{\mathrm{COP}}$ (cm) is the COP lateral displacement, ${\mathrm{LongitudinalD}}_{\mathrm{COP}}$ (cm) is the COP longitudinal displacement, ${\mathrm{Dis}}_{\mathrm{COP}}$ (cm) is the COP displacement, ${\mathrm{Speed}}_{\mathrm{COP}}$ (cm/s) is the shift speed of COP, and Δt is the time interval.

## 3. Results and Discussions

**Figure 4:**
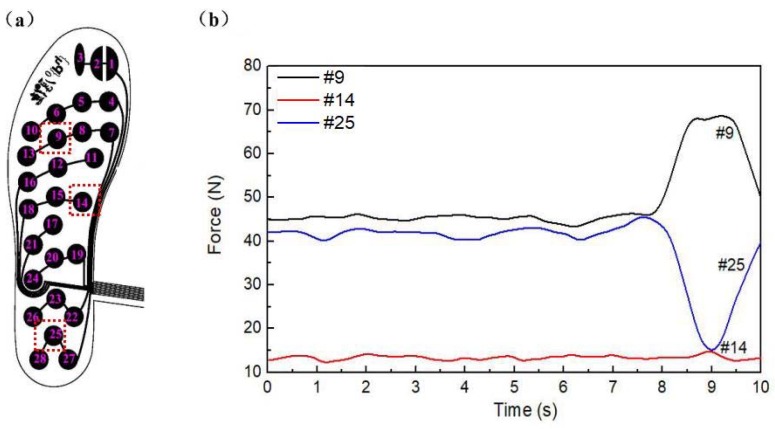
(**a**) Sensor distribution of plantar pressure insoles; (**b**) Pressure variation of characteristic points in plantar.

As shown in Figure 4, the pressure change curve was acquired from characteristic points of the insoles during standing. The points of Nos. 9, 14, and 25 were taken from the forefoot, midfoot, and rearfoot, respectively. In the static positions of a relaxed standing posture, the forefoot and rear foot were loaded with a large force, while the midfoot was almost unloaded. As shown in Figure 4, the forces of point 9 and 25 were much larger than that of point 14 at midfoot, which was consistent with the actual foot pressure distribution. In order to show the dynamic characteristic, the subject was asked to do a tilting motion at 8 s. Therefore, there was a change at 8–10 s for point 9 and point 25, which was caused by the transition from the standing to the forward leaning posture. In the forward tilt, the midfoot is almost unstressed, so point 14 had no significant change. The change of force indicated that the characteristic point of insoles had a sensitive response to foot pressure.

**Figure 5:**
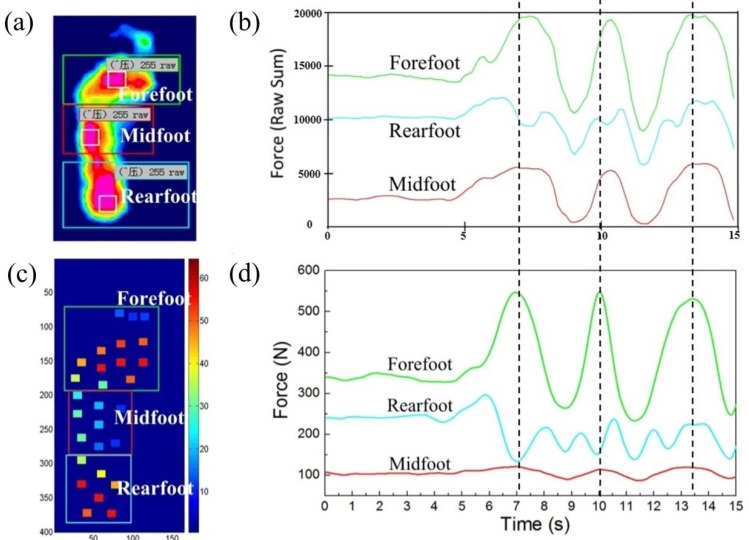
Pressure variations of forefoot, midfoot, and rearfoot regions during standing. (**a**) The plantar pressure distribution image obtained from Tekscan; (**b**) Pressure curves measured using Tekscan; (**c**) The plantar pressure image obtained from insole; (**d**) Pressure curves measured using insoles.

The regularity of foot pressure in the forefoot, midfoot, and rearfoot regions was investigated with the insoles and compared with the pressure distribution measured using Tekscan. As shown in Figure 5, the results from two types of equipment were basically the same. In Figure 5d, the force in the midfoot region was lower than that in Figure 5b, which may have been caused by the relatively lower sensor element density of the insole. At 7 s, both forefoot forces appeared as a wave peak and the midfoot ones appeared as the valley. Although the change was not obvious, the same trends occurred at the 10 s and 13.4 s.

To investigate the validity and reliability of the pressure insole, we collected the data of 12 healthy subjects with different body weights over 30 s each and compare them with the pressure value of Tekscan sensors at the same position. Each subject wore the pressure insole and stood on the Tekscan pressure pad, and data from the two devices was collected simultaneously; the mean pressure data over 30 s was analyzed. This experiment was based on the theory of Pearson correlation in which the Pearson correlation coefficient (r) can reflect the linear correlation between two sets of vectors. Thus, the validity could be conducted in accordance to the r between the two sets of the pressure force. The pressure force of each sensing point of five subjects is shown in Table 1. The Pearson correlation coefficients of the corresponding points for each subject was 0.8820, 0.8705, 0.8372, 0.7535, and 0.7443; the Pearson correlation coefficient of the other seven subjects were 0.8186, 0.8808, 0.6704, 0.8452, 0.7682, 0.7749, and 0.7521, and the mean value of the 12 subjects was 0.7934, which revealed a strong correlation between the two devices.

**Figure 6:**
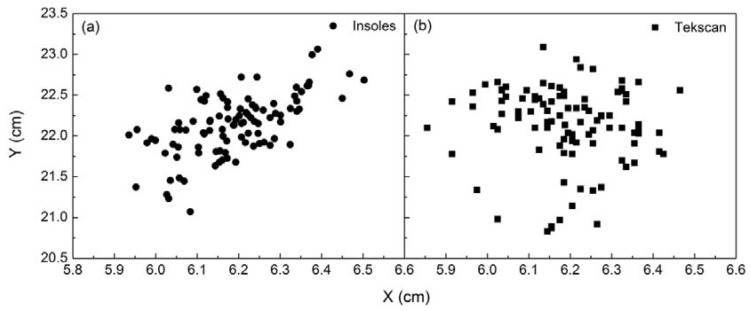
(**a**) Distribution of COP measured using insoles; (**b**) Distribution of COP measured using the Tekscan system.

The distribution of COP at standing posture measured using pressure insoles and the Tekscan system is shown in Figure 6a,b, respectively. As can be seen from the figure, in the case of standing, the X-coordinate of COP measured using insoles was within the range of 5.93–6.50 cm, and the coefficient of variation was 0.019; the Y-coordinate was within the range of 21.07–23.06 cm, and the coefficient of variation was 0.016. The X-coordinate of COP measured using the Tekscan system was within the range of 5.85–6.46 cm and the coefficient of variation is 0.020; the Y-coordinate was within the range of 20.82–23.08 cm and the coefficient of variation was 0.021. The coefficients of variation of COP in the X- and Y-directions measured by the two systems were similar, demonstrating a reliable consistency between the insoles and Tekscan.

**Figure 7:**
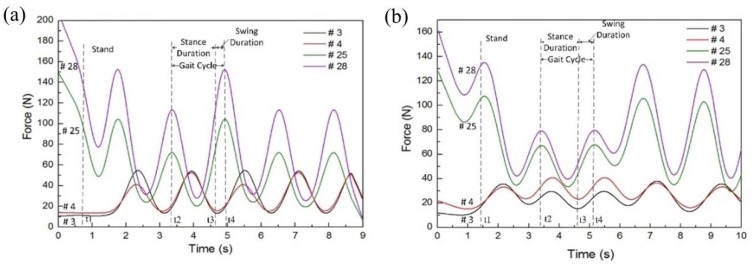
Pressure variation at characteristic points of left and right foot during five normal walking gait cycles. **(a)** The pressure curve of left foot during walking; **(b)** the pressure curve of right foot during walking.

Subsequently, the dynamic gait experiment could be carried out, and the relative gait parameters were analyzed. As shown in Figure 7, the pressure had obvious fluctuation at the characteristic points of the left and right feet during five gait cycles of normal walking. A gait period was defined as the time between two heel landing phases, and one gait cycle was divided into two stages: the stance phase, which was the time between the heel landing and the tiptoe lifting, and the swing phase, which was the time between the tiptoe lifting and the heel landing again. In Figure 7, t2 is the time of heel landing, t3 is the moment of tiptoe lifting, t4 is the time of heel landing again, and a full gait cycle was obtained from t2 to t4. At time point t2, the forces of points 25 and 28 reached the maximum value because of the heel landing on the floor. At time point t3, the tiptoe was off the ground, thus the forces of points 3 and 4 reached the minimum. Gait experiments have shown that the pressure insole can also collect pressure data during dynamic processes. A summary of gait data is shown in Table 2.

Four healthy subjects in normal walking have been tested using the insoles and Tekscan system, and the time of the stance phase, swing phase, and gait cycle have been extracted for comparison. There was no significant difference between these two systems. The maximum time difference in stance phase duration was 0.130 s, and the minimum value was zero. The maximum time difference in swing phase duration was 0.161 s and the minimum was 0.033 s. The difference in the gait period was within the range of 0.098 s to 0.028 s. Furthermore, the cosine similarity of the stance duration, swing duration, and gait cycle time of the left and the right foot between the two devices were 99.97%, 99.34%, 99.97%, 99.91%, 99.04%, and 99.98%, respectively. The results of these static and dynamic experiments demonstrate that the pressure insoles can measure dynamic plantar pressure distribution and it may be functional enough to be used for balance assessment. Therefore, the developed pressure insoles have been employed in the following study.

**Figure 8:**
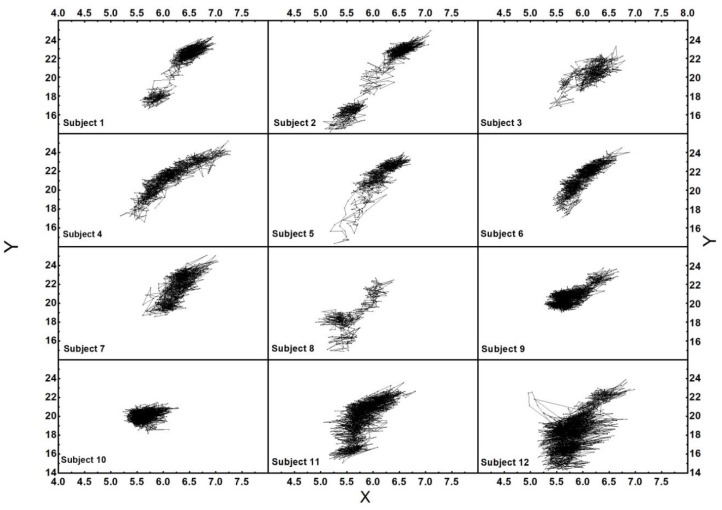
The COP trajectory of 12 subjects.

The balance assessment has also been carried out using the apple picking game with the pressure insoles and Kinect sensor, as displayed in Figure 3c. The plantar pressure data of 12 subjects were extracted and the relative parameters were calculated using Equations (2)–(7). Figure 8 shows the COP trajectory of 12 subjects during the experiment. The balance ability of each subject could be assessed using the degree of the COP displacement. On the premise of completing the experiment under the condition of external disturbances, the degree of dispersion of the COP distribution can reflect a person’s adjustment ability to maintain dynamic equilibrium. Some subjects completed the experiment with non-significant COP displacement, indicating that they can reach equilibrium more quickly with an excellent capability of self-adjustment balance. As shown in Figure 8, the Y-displacement of COP of the 12 subjects were 7.64, 10.71, 6.57, 8.61, 9.66, 7.36, 6.53, 7.77, 4.70, 3.34, 8.53, and 8.48 cm, respectively. Although there were significant differences in the COP displacement and dispersion of the COP distribution of the 12 subjects, they all had equally good general balance ability and self-adjustment ability. The results prove that the balance assessment system can obtain different COP displacements of 12 subjects.

In the designed Kinect game, we mainly aimed to test the balance ability in the anterior direction. The apples in the virtual scene were located within 1–2 m on the front side of the human body, as show in Figure 1b. In a walking gait cycle, the trajectory of the COP moved from the heel to the forefoot, and the maximum displacement of the anterior–posterior direction (Y-displacement) was close to the foot length in theory and the X-displacement was the same as the foot width in theory. In the experiment, the subjects stood on the ground and leant the body forward to pick the apples, so there was no process from a heel-strike landing to a forefoot landing. Therefore, the maximum Y-displacement of the COP in the experiment was equal to or smaller than that of normal walking. The Y-COP displacement during the balance experiment was measured and normalized for the foot length to obtain the Y-proportion [30,31]. We mainly investigate the Y displacement of each subject in the following study to assess the anterior–posterior balance ability [26].

The calculation result of the balance experiment is shown in Table 3. The X-proportions of most subjects were close to one and there was no significant difference in the X-displacement, while the Y-displacement had an obvious difference between the subjects. The Y-proportion of the 12 volunteers were 0.126–0.404. The mean value of the Y-proportion of 12 subjects was 0.281. The standard deviation was 0.076. Therefore, there was little difference in the balance ability of healthy people. They have enough ability to control the body balance to ensure the completion of the test.

**Figure 9:**
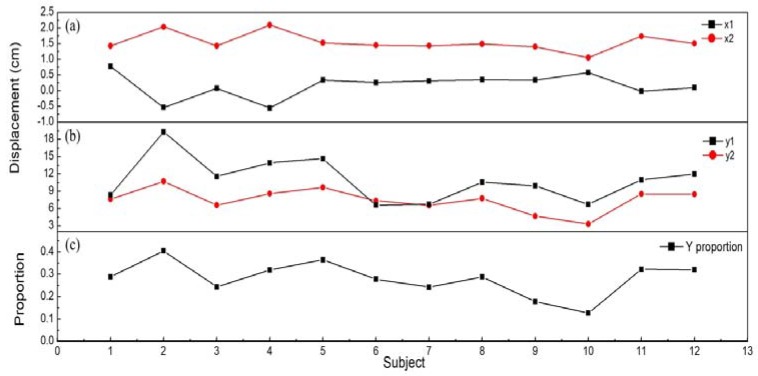
The maximum displacements of COP and the Y-proportion obtained from two experimental methods. (**a**) The X-displacement of 12 subjects, (**b**) The Y-displacement of 12 subjects. X1 and Y1 are the data from the STS test and X2 and Y2 are the data from the balance experiment. (**c**) The Y-proportion of 12 subjects.

Subsequently, we performed the STS test, where each subject conducted the STS test three times, and the mean value of the maximum COP displacement was taken for analysis. The results were compared with that of the balance experiment to verify the effectiveness of the method proposed in our study. Figure 9 shows the Y-proportion, and the maximum Y- and X-displacements of the 12 subjects obtained by the two methods. In the STS test, the X-displacements of 12 subjects were almost constant with no variation and all data points are displayed as the X1 curve in Figure 9a. Because the subjects only moved backwards and forwards during standing up and sitting down, X-displacement changed very little [32]. In the balance experiment, the X2 curve varied erratically. The X-displacements of most subjects were around 2 cm, and larger X-displacements were produced in some cases, which arose from the Kinect guided balance adjustments in the anterior–posterior and medial–lateral directions during the experimental process.

The curves Y1 and Y2 in Figure 9b show a positive correlation, which were calculated using correlation analysis, and the Pearson and Spearman correlation coefficients reached 0.7516 and 0.8182 (0.5 to 0.8 indicates a positive correlation, 0.8 to 1.0 indicates a high positive correlation). Figure 9c shows the Y-proportion of the 12 subjects. The Pearson and Spearman correlation coefficients between Y-displacement and Y-proportion were 0.9619 and 0.9720. The smaller Y-proportion meant better balance. The smaller COP oscillation was produced by a person with good balance to succeed in the task of lifting up from the chair [33,34,35]. Therefore, the smaller the Y-displacement was changed in the STS test, the smaller the Y-proportion changes will be obtained in the balance experiment. After the verification by the STS test with a series of experiments, it showed that the proposed method could complete a certain evaluation in the forward and backward directions.

**Figure 10:**
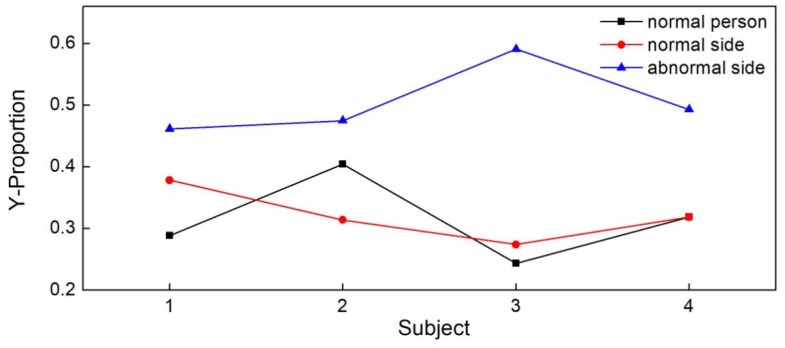
The Y-proportion of four normal subjects with the normal side and the abnormal side of four subjects with a right leg sports injury.

Finally, we conducted experiments on four abnormal volunteers, each with a right leg sports injury, to compare the experimental data of the abnormal side with the normal side and normal subjects, and the results are shown in Figure 10. The Y-proportions of the abnormal side were larger than that of the normal side and the normal subjects. The mean value of the Y-proportion of the abnormal side and normal side were 0.505 and 0.321, respectively. The Y-proportions of the normal side had no significant difference compared to normal subjects. The results demonstrate that there was a decided difference of the balance ability between the normal side and the abnormal side of subjects.

The design of the balance assessment experiment was based on the fact that the upper limb movement would induce balance disturbance in the two-legged standing situation. The 12 volunteers had no limb disease, the two-legged state was relatively balanced, and the two-footed pressure center trajectory and other parameters were basically the same. Therefore, we only studied the experimental data of one foot to evaluate the balance of healthy subjects. Finally, we performed a balance experiment on the abnormal subjects. The foot pressure data on the normal side and the abnormal side were analyzed and compared for significantly different dates to demonstrate the effectiveness of the proposed method.

We mainly analyzed the postural stability of the subject in the anterior–posterior direction. When the human body adjusts the posture balance, there will be anterior–posterior and medial–lateral adjustment. When the same motion experiment is performed, the subject has a larger X–Y displacement. A positive correlation was obtained through the correlation analysis of the balance experiment and STS test results. The experimental results of abnormal people also showed significant differences between the normal side and the abnormal side. Therefore, the proposed system and evaluation method proved that they could reflect the balance ability to a certain extent.

On the other hand, although this work mainly analyzed the Y-displacement, the X-displacement can also reflect the balance ability. In the anterior–posterior direction, the balance can be maintained through moving forward or backward when it is impossible to keep a static balance. The medial and lateral direction imbalance is more difficult to return to equilibrium by moving left or right and so many older people have lateral falls, leading to an increase in the risk of hip fractures. Thus, the study of medial and lateral direction imbalance is also of great significance. The human body adjusts the balance in different directions, so it cannot be judged by the single direction. The change of the single direction can only reflect the balance ability to a certain extent. Therefore, it will be a study of the ability to balance in multiple directions in the future.

## 4. Conclusions

In this study, the quantification of dynamic balance ability has been proposed and tested on 12 healthy subjects and 4 abnormal subjects with one side leg sports injury using the Kinect sensor and pressure insoles. The balance experiment of the measured subject was realized by analyzing the COP displacement under the situation of the Kinect guided game. The results were closely correlated with the STS experimental results. Our experiment results demonstrated that the proposed method based on the Kinect sensor and pressure insoles could perform the balance assessment well, and it displayed a valuable result of the strength of dynamic balance. Moreover, the novel evaluation process has higher repeatability and can be widely applied in the future.

## Figures and Tables

**Table 1:** The pressure data obtained by two pressure measurement systems.

Point	Subject									
	1		2		3		4		5	
	Insole ^a^	Tek ^b^	Insole	Tek	Insole	Tek	Insole	Tek	Insole	Tek
1	26.22	150	19.23	179	17.24	135	25.22	187	15.25	147
2	13.77	78	15.25	144	5.88	30	12.78	122	4.89	45
3	13.77	66	9.81	71	5.88	30	5.88	37	12.78	115
4	25.22	141	11.29	119	19.23	172	11.29	102	18.23	168
5	12.78	96	27.22	218	13.77	113	12.78	150	12.78	119
6	20.72	112	20.72	137	13.77	113	13.77	140	13.27	120
7	31.75	165	31.24	245	27.72	191	31.24	161	24.22	199
8	45.44	185	27.72	223	32.75	255	41.37	242	16.74	132
9	45.95	202	45.95	252	44.93	255	44.93	255	12.78	124
10	27.22	188	13.77	102	27.22	199	25.22	196	13.77	134
11	25.22	126	19.23	194	25.22	179	26.22	191	9.32	68
12	20.72	104	15.75	124	20.72	133	13.77	137	5.39	51
13	49.54	212	45.95	240	51.59	220	58.81	214	12.78	120
14	19.23	90	11.29	91	13.23	102	11.29	98	10.31	81
15	13.77	67	5.88	46	13.77	91	13.77	208	9.32	74
16	27.22	187	13.77	136	27.22	219	27.22	154	9.81	79
17	5.88	50	13.77	136	12.78	79	12.78	146	5.88	48
18	25.22	187	12.78	134	25.22	207	19.23	204	5.88	39
19	15.25	84	11.29	108	18.23	165	13.27	139	16.24	167
20	12.78	73	12.78	132	13.77	119	13.77	147	13.77	113
21	27.22	168	13.77	156	26.22	237	19.23	164	7.84	84
22	27.22	168	27.22	255	27.22	208	29.73	234	24.22	193
23	41.37	255	40.35	255	41.87	255	41.37	226	28.73	255
24	24.22	110	12.78	119	25.22	192	25.22	218	13.77	160
25	36.8	255	36.8	255	45.95	255	38.83	255	100.51	255
26	43.4	205	41.37	213	50.56	205	41.37	204	44.93	225
27	31.24	207	29.23	238	27.22	206	27.22	193	48.51	225
28	51.59	229	36.8	176	45.44	210	52.62	209	125.58	255
r	0.882		0.8705		0.8372		0.7535		0.7443	

^a^ The unit of pressure insole is N; ^b^ Tek: The Tekscan system, arbitrary unit.

**Table 2:** The gait data obtained using two pressure measurement systems.

		Pressure Measurement System			The Pressure Insole		
	Foot	Stance Phase (s)	Swing Phase (s)	Gait Cycle (s)	Stance Phase (s)	Swing Phase (s)	Gait Cycle (s)
subject 1	L	1300	0.300	1600	1300	0.267	1567
	R	1260	0.450	1710	1213	0.399	1612
subject 2	L	1300	0.350	1650	1344	0.249	1593
	R	1460	0.350	1810	1501	0.281	1782
subject 3	L	1300	0.330	1630	1373	0.227	1600
	R	1390	0.400	1790	1520	0.239	1759
subject 4	L	1290	0.310	1600	1370	0.270	1640
	R	1500	0.420	1920	1550	0.337	1887

**Table 3:** The balance experiment data.

Subject	X-Displacement (cm)	Y-Displacement (cm)	The Shift Speed of X (cm/s)	The Shift Speed of Y (cm/s)	X-Proportion	Y-Proportion
1	1427	7638	0.157	0.495	1223	0.288
2	2040	10.712	0.163	0.480	1359	0.404
3	1429	6574	0.110	0.406	0.947	0.243
4	2096	8606	0.127	0.379	1361	0.319
5	1527	9661	0.124	0.390	0.819	0.365
6	1450	7356	0.126	0.343	0.849	0.278
7	1433	6535	0.148	0.480	0.821	0.242
8	1489	7767	0.146	0.372	0.812	0.288
9	1402	4701	0.157	0.409	0.807	0.177
10	1055	3336	0.165	0.392	0.647	0.126
11	1738	8527	0.190	0.472	1.009	0.322
12	1506	8482	0.186	0.465	0.938	0.320

X-displacement: The X-displacement of the COP; Y-displacement: The Y-displacement of the COP; the shift speed of X: The shift speed of X-displacement; the shift speed of Y: The shift speed of Y-displacement; X-Proportion: The proportion is obtained by dividing the X-displacement with the foot width; Y-Proportion: The proportion is obtained by dividing the Y-displacement with the foot length.
